# Data visualisation to support obesity policy: case studies of data tools for planning and transport policy in the UK

**DOI:** 10.1038/s41366-018-0243-6

**Published:** 2018-11-23

**Authors:** Pablo Monsivais, Oliver Francis, Robin Lovelace, Michael Chang, Emma Strachan, Thomas Burgoine

**Affiliations:** 1 UKCRC Centre for Diet and Activity Research (CEDAR), MRC Epidemiology Unit, University of Cambridge School of Clinical Medicine, Institute of Metabolic Science, Cambridge Biomedical Campus, Cambridge, CB2 0QQ UK; 2 Department of Nutrition and Exercise Physiology, Elson S. Floyd College of Medicine, Washington State University, Spokane, WA 99210 USA; 3 Institute for Transport Studies, University of Leeds, Leeds, LS2 9JT UK; 4 Town & Country Planning Association, London, SW1Y 5AS UK; 5 Carnegie School of Sport, Leeds Beckett University, Leeds, LS6 3QT UK; 6 Health Improvement, Leeds City Council, Leeds, LS2 9JT UK

## Abstract

Data visualisation is becoming an established way to drive discovery and develop theory and hypotheses among researchers. Data visualisations can also serve as tools for knowledge translation with policy makers, who are increasingly using data and evidence to inform and implement policy. For obesity policy, data visualisation tools can help policy makers and other professionals understand the socio-spatial distribution of risk factors and quantify social and environmental conditions that are recognised upstream determinants of diet, activity and obesity. The demand for and use of data visualisation tools can be driven by an identified policy need, which can be met by researchers and data scientists. Alternatively, researchers are developing and testing data visualisations, which may be subsequently adapted for, and adopted by policy users.

Two recently-released interactive data visualisation tools in the UK illustrate these points. The Propensity to Cycle Tool (PCT) was developed with funding from the UK government to inform the investment of cycling infrastructure in England. The Food environment assessment tool (Feat) evolved as a translational output from a programme of epidemiological research. This article uses PCT and Feat as case studies, drawing parallels and contrasts between them. We discuss these two tools from policy context and scientific underpinnings, to product launch and evaluation. We review challenges inherent in the development and dissemination of data tools for policy, including the need for technical expertise, feedback integration, long-term sustainability, and provision of training and user support. Finally, we attempt to derive learning points that may help overcome challenges associated with the creation, dissemination and sustaining of data tools for policy. We contend that, despite a number of challenges, data tools provide a novel gateway between researchers and a range of stakeholders, who are seeking ways of accessing and using evidence to inform obesity programs and policies.

## Background

Obesity is both a public health crisis and a scientific challenge. Within the last 40 years, the prevalence of obesity in the UK has nearly quadrupled, from 6–8% of the adult population in 1980 to 26% today [1, 2], costing to the public purse in England an estimated £27 billion per year [3]. The volume of obesity research has undergone an even more dramatic increase over the same period, from roughly 1200 articles published in 1980 to over 21,000 articles published in 2017 alone. This scientific momentum has brought with it innovation of data sources and analytical approaches. While the visual representation of data, for instance, in charts and other graphics, has long been integral to the scientific process [4], scientific innovations and use of ‘big data’ in particular have driven new ways of visualising data. Data visualisations are important for communicating to scientific audiences, and increasingly an essential component of disseminating complex research findings to the public, and helping to better inform policy development. Moreover, the urgent societal implications of obesity are challenging researchers to carefully consider the potential of data visualisations to better serve public policy and practice.

### What are data visualisations?

Data visualisation is commonly defined as a graphical approach to the presentation of data. Data visualisation can make data more accessible by providing an opportunity to examine and explore large amounts of often complex, quantitative information at once [5]. Although this broad definition of data visualisation includes scientific illustrations and figures, these are targeted at academic or otherwise specialist audiences, and so do not realise the full potential of data visualisation, which also includes interactivity and flexible outputs for users. Furthermore, some researchers, particularly those from computing- and graphics-intensive disciplines, have advocated the use of data visualisations not just as an output or adjunct of research but as a complement to traditional statistically-based approaches to exploring data, developing theory and testing hypotheses [6].

### Data visualisation for obesity research

Since the publication of the Foresight report in 2007 [7], there has been growing recognition that obesity results from the interplay of multiple biological, behavioural and social determinants within a complex system [8, 9]. The complex, multifactorial nature of population-level obesity poses a challenge to the prevailing theoretical and analytic paradigms commonly used in human obesity research [10].

**Fig. 1:**
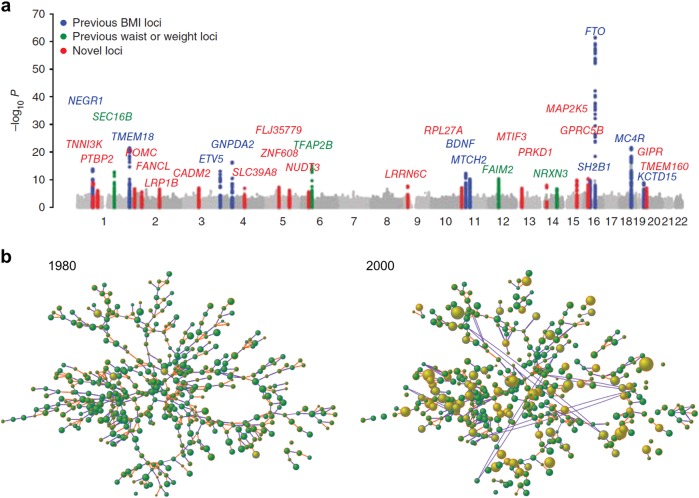
**a**, **b** Examples of data visualisations used in population-level obesity research. A Manhattan plot used for identifying genetic loci associated with obesity (**a**) (From reference [12], reprinted with permission of the authors.) A network diagram for identifying social relationships among obese and non-obese members of a community (**b**) (From reference [13], reprinted with permission of the authors.)

In obesity epidemiology, statistical approaches to understanding associations and putative causal factors have been increasingly complemented by visual approaches. For example, among biological determinants of obesity, genetics account for 40–70% of the population variance in obesity susceptibility [11]. Genome-wide association studies, visualised with Manhattan plots (Fig. 1a), have allowed researchers to identify genetic variants at numerous loci that are strongly associated with obesity and common metabolic diseases [12]. Obesity also has strong social determinants, and social scientists have used Barabasi–Albert network visualisations (Fig. 1b) to identify clusters of obese adults within a social network, and over time [13]. These two examples illustrate the usefulness of data visualisation to systematise and explore large amounts of empirical ‘big data’. Data visualisation can also facilitate knowledge exchange and translation for policy [14].

### Data visualisation for policy

Big data and other large, routine data sets have the potential to support decision-making and policy, particularly when presented geographically [15]. Data to inform public health priorities and action at local and smaller geographic levels are increasingly available. In the UK, data visualisations and other data-based tools feature prominently in Public Health England’s (PHE's) *Knowledge Strategy* [16]. The development and dissemination of such tools, in addition to the standardisation, structuring and linkage of the data and systems underlying them, provides a means of supporting decision making and informing policy and practice across public health [16]. For example, *Fingertips* [17] provides a public access interface for data on disease burden, risk factors and other population health indicators for England, at various levels of geographic specificity. Data on obesity prevalence in the adult population can be retrieved and stratified by a number of sociodemographic characteristics, as well as reported and displayed geographically [18]. The platform also allows users to compare prevalence for one group or within one area against the national average or against other groups or areas. Some regions in the UK are developing similar capabilities. The Leeds Observatory hosts a wide range of economic, social and health indicators for the region, at various levels of geography [19].

An important feature of both Fingertips and the Leeds Observatory tools is their capacity to display data in tabular format as well as in a range of graphics that facilitate comparisons between social groups and between regions. These graphics include choropleth maps (spatial data visualisations), which use colour or shading to convey differences in disease rates or risk factors in a way that is easily recognisable and intuitive. Spatial data visualisations can facilitate analysis at a range of geographic scales that are relevant for policy, for example by providing estimates at the level of local government administrative boundaries. Whilst available to everyone on the Internet, Fingertips is intended to help local authorities identify populations and areas that may benefit from intervention.

### Data for obesity policy

Four important trends have converged to increase the importance of local data for decision-making for obesity prevention within local government. First, with the Health and Social Care Act of 2012, the responsibility for public health moved from the National Health Service’s Primary Care Trusts into local governments, which were given the authority to set priorities and policy independently [20]. Second, both local and national policy makers are increasingly complementing individually-targeted obesity programs with population-level interventions that are local context specific [21, 22]. Thirdly, the National Planning Policy Framework, which sets the overall planning guidance for local government in England, requires local health needs to be taken into account when developing planning policies [23]. Finally, there has been an increasing scientific focus on upstream determinants of disease (including obesity), which often vary locally [9]. Moreover, there is a growing recognition of the multiple avenues by which population-level determinants of obesity can be addressed, including through cross-sectoral policy, systems and environmental action [24], including planning and transport policy.

Planning and transport policies are two promising areas relevant for obesity because they represent the potential to build and modify aspects of the built environment that can improve population diet and physical activity. Since the 1990s, efforts to promote active travel have been part of the UK national transportation strategy, with a focus on reducing congestion, improving air quality and improving accessibility [25]. While the national approach to transport planning continues to be set by central government, specific decisions about pedestrian and cycle infrastructure are being made by local authorities. With regards to diet, local governments have long been responsible for considering planning applications for the establishment of food retail outlets, but in the last 10 years, this authority has come to be seen as a possible point of public health intervention, with the potential to improve the healthfulness of the food environment. For example, both local governments and PHE have advocated for the use of planning powers to limit the proliferation of fast-food outlets in towns and cities [26].

Historically, there has been little in the way of easily accessible data available to guide local decision making with the potential to encourage active transport and healthier dietary behaviour. Below, we outline two case studies, of tools recently developed by ESRC Strategic Network for Obesity members at the Centre for Diet and Activity Research (CEDAR), which use spatial data visualisation as a platform to support local decision making. After briefly describing each tool, the main aim of this manuscript is to draw from these case studies examples of generalised learning experiences and challenges, while highlighting important implications for research and policy. The key characteristics of these tools, including brief technical details, are presented in Table 1. This paper is not intended to provide a detailed technical description of each tool. For further description of the more technical aspects of PCT, see Lovelace et al. [27]. Technical aspects of Feat will be described in a forthcoming publication.

**Table 1:** Key characteristics of two data visualisation tools for policy

	Propensity to Cycle Tool (PCT)	Food environment assessment tool (Feat)
**Administrative**		
URL	www.pct.bike	www.feat-tool.org.uk
Public launch	May 2015 (prototype) March 2017 (official launch)	July 2017
Region(s) covered	England, Wales	England
Funding source(s)	UK DfT	ESRC Impact Acceleration Account and University of Cambridge MRC Epidemiology Unit
Externally commissioned?	Yes, by UK DfT	No
Hosting institution	Mythic Beasts via Cambridge, Westminster and Leeds Universities	MRC Epidemiology Unit, University of Cambridge
Update frequency	Approximately yearly	Quarterly
Historic data available	No	Yes
**Technical**		
Key output data	Cycle network maps	Density and mix of food outlets
Software	R (packages: shiny, leaflet, stplanr)	ArcGIS, Leaflet
Basemap	Open Street Map	Open Street Map
Geographic display levels	Area (MSOA, LSOA), Desire line, route, street network	County, Local authority, MSOA, LSOA, ward, unit postcode
Input environmental data, source	OpenStreetMap; Origin–Destination data, routing from CycleStreets.net	Food outlets, Ordnance Survey Points of interest; geographic boundaries, UKBORDERS and Ordnance Survey Code-point with Polygons
Input behavioural data, source	UK Travel Survey, Dutch Travel Survey	N/A
Other data, source	Population, 2011 UK census	Population, 2011 UK census
User modifiable through open source code?	Yes, https://github.com/npct/pct-shiny	No
**Other**		
Data access	Free data access of all levels in multiple formats	Free data access of all levels in map format only
User support contact	pct@pct.bike	feat-tool@mrc-epid.cam.ac.uk

## Case study 1: The Propensity to Cycle Tool

Active travel (walking and cycling) is an important source of routine physical activity that can be integrated into everyday life as part of a wider sustainable transport system. However, in many countries, private motor vehicles are still the default option, even for short trips, which make-up the majority of trips in countries such as the UK, where the majority (56%) of car journeys driven are less than 5 miles [28]. Cycling is a particularly promising transport mode in this context because it has the potential to replace more car trips than walking, with an average distance of 3.5 miles for cycle trips in 2016 compared to an average of only 0.7 miles for walking trips.

Nevertheless, the prevalence of cycling for transportation in the UK is low, with approximately 3% of journeys being undertaken by bike [29]. International experience shows that high-quality infrastructure can play a key role in promoting cycling uptake [30]. Separated cycle paths, for example, in addition to other elements of cycling infrastructure, have been found to be associated with an uptake of cycling for commuting. But where should this infrastructure be built to yield the greatest impact? It is in this context that the UK’s Department for Transport funded development of the Propensity to Cycle Tool (PCT), to provide a publicly-accessible and local evidence-base for planning strategic cycle networks based on routes, corridors and ‘desire lines’ with high potential for cycling uptake [27, 31].

The project can be divided into three main stages: software testing and development; data analysis; and national deployment. In the development phase, researchers from four universities (Leeds, Cambridge, Westminster and London School of Hygiene and Tropical Medicine) collaborated to develop a prototype web application using Shiny, an R package for interactive visualisation [32]. An additional R package was developed for geographical processing and routing on the road network of the input data: origin–destination pairs between commute zones from the 2011 Census. Noteworthy features of the development process were the use of GitHub for code hosting, version control and communication, and the use of ‘continuous integration’ on a test server so all collaborators could comment on the latest version of the tool as it evolved.

**Fig. 2:**
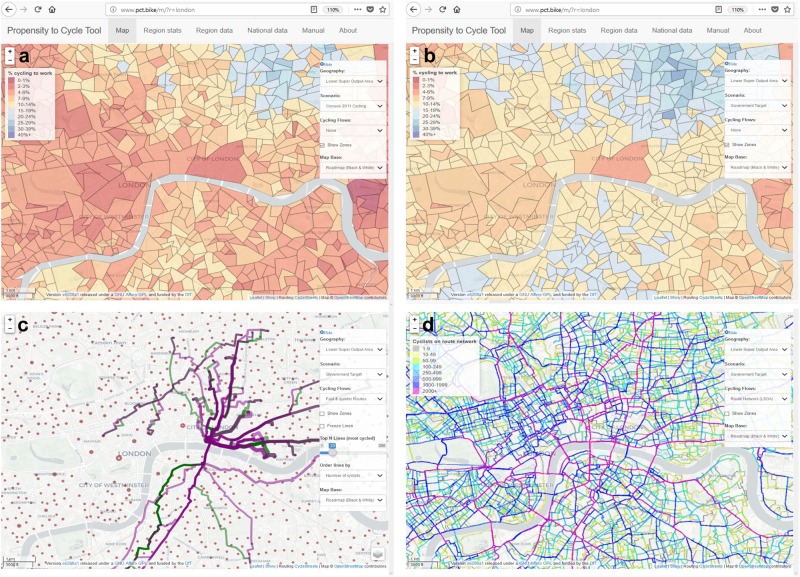
Screenshots of the PCT show four visualisation layers for central London. Panels (a) and (b) show two scenarios, ‘Census 2011 Cycling’ and ‘Go Dutch’ at the small area (LSOA) level. These highlight areas in need of investment in the short-to-long term based on current trip patterns. Panel (**c**) shows the same area but with the *Fast* and *Quieter Route* layer activated. Panel (d) shows the route Network layer at the LSOA level, the most geographically detailed layer in the PCT

In the data analysis stage, scenarios were developed via in-depth analysis of National Travel Surveys in the UK and the Netherlands [31]. The scenarios are an important element of the PCT, allowing stakeholders to visualise what a shift to cycling could look like, in terms of number of commuter cyclists using different parts of a (yet-to-be-built) network of protected cycleways taking direct routes to major employment centres. Figure 2 shows four visualisation layers in PCT for central London. Figure 2a and 2b show two scenarios, ‘Census 2011 Cycling’ and ‘Go Dutch’ at the small area (LSOA) level. These highlight areas in need of investment in the short-to-long term based on current trip patterns. Figure 2c shows the same area but with the *Fast* and *Quieter Route* layers activated to highlight existing routes between the most popular desire lines for cycling under the Government Target scenario, and where they go. Figure 2d shows the Route Network layer at the LSOA level, the most geographically detailed layer in the PCT, which can inform investment in cycle networks down to the street network nationwide.

The final deployment phase was the most important in terms of policy impact. Unusually for an academic project, this involved setting-up a physical server with a dedicated web hosting company and hiring an independent web developer. Before the PCT was officially launched in Spring 2017, a process of user feedback was used to improve the tool for the target audience of local authorities, which included approximately 100 test-users at 12 user-testing workshops held at key events, such as Cycle City Active City (CCAC). These users provided feedback that was incorporated into the version that was later launched.

After launch, the PCT has already begun to realise impact; we know of four local authorities (Transport for Greater Manchester, Merseyside, Warrington, Exeter) who have designed strategic cycle networks based at least in part on the results of the PCT, and it has enabled cycling advocates and other local stakeholders to be engaged in the debate, using high-quality information, to envisage healthier transport systems across the UK.

## Case study 2: The Food environment assessment tool

Neighbourhood food environments—the distribution, density and mix of accessible food outlets—are a recognised influence on what we eat, our body weight and health [3]. As a result, published guidelines from PHE (PHE), the Local Government Association (LGA) and the Greater London Authority (GLA) provide strong support for local authorities to, for example, influence the food environment to promote and support healthier food choices [26]. In 2017, PHE also released their ‘*Out of home food provision toolkit*’ as part of a population-level approach to obesity prevention [22]. The toolkit cites the need to take local action, and to understand the local food environment. A determining factor in the development of effective policy is strong supporting evidence.

While health researchers have developed a number of methods to assess the food environment, little of this knowledge has been translated for the purpose of environmental assessment by practitioners and policymakers. Some local authorities have conducted one-off, bespoke food environment assessments locally [33], but a comprehensive platform for objective, nationwide surveillance of food access that can be used both nationally and locally, has been critically lacking in England.

**Fig. 3:**
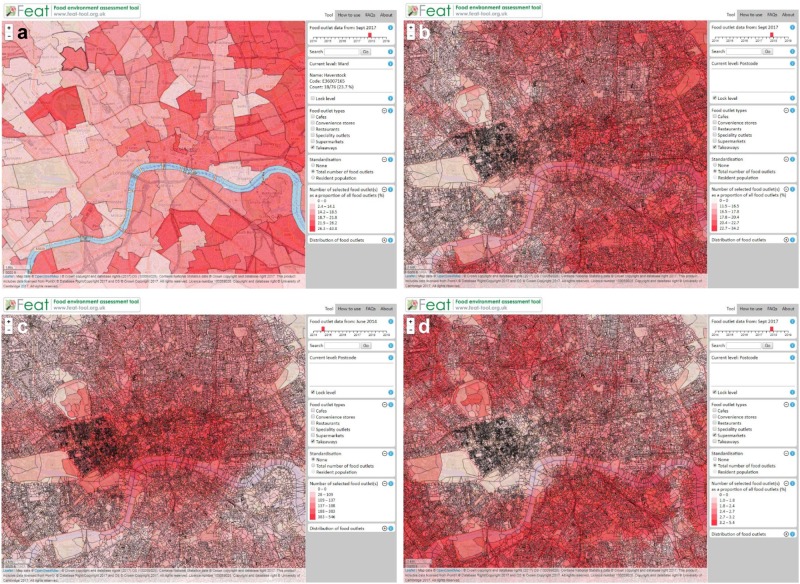
Illustration of Feat in action in central London: **a** the user has selected to display an electoral ward level estimate of takeaway food outlet number, as a proportion of all food outlets, for September 2017; **b** the user has selected to display a postcode level estimate of takeaway food outlet number, as a proportion of all accessible food outlets, for September 2017; **c** the user has selected to display a postcode level estimate of takeaway food outlet number (unstandardised, raw counts), for June 2014; **d** the user has selected to display a postcode level estimate of supermarket number, as a proportion of all accessible food outlets, for September 2017

The Food environment assessment tool (Feat) was developed to address this need, informed by research evidence. Feat is an interactive, web-based resource for mapping, measuring and monitoring regional and neighbourhood food access across England and over time (see Figure 3). Developed with funding from the Economic and Social Research Council’s Impact Acceleration Account, along with in-kind funding from the MRC Epidemiology Unit at the University of Cambridge, Feat was intended to translate the findings of our state-of-the-art scientific evidence, for use by those in planning, environmental and public health within local authorities, regional and national public health bodies.

Feat was developed by an interdisciplinary team of researchers and technicians with expertise in epidemiology, geography, data management, web-based programming and knowledge exchange, based entirely within a single academic department. Feat was developed through an iterative process of creating and releasing a minimum viable product for testing, receiving feedback (including further canvassing of demand), and refining before releasing the next version. At no point was the development team hoping to release the perfect version of Feat; only one that was good enough to be released, tested and learned from—an approach endorsed in UK policy approaches to online service delivery [34]. The first fully-functional version of Feat (Feat alpha) was launched in April 2016, exclusively to multiple groups of stakeholders from across policy and practice in England. Each of the stakeholder groups, including 63 representatives from across local and national public health and 22 local authorities, agreed to test and evaluate Feat alpha for a period of 6 weeks, and provide structured feedback via email questionnaire. In addition, we sought engagement with key stakeholders through showcasing the Feat alpha in person at several local governments, PHE, and at PHE conferences and other meetings with majority policy and practice audiences.

The feedback we received from this process was used to evaluate Feat’s current and potential functionality. The feedback—in the form of Likert and free-text responses, as well as workshops to identify priorities—allowed us to schedule updates with respect to stakeholder demand, culminating in the development of a Feat beta. We also used this process to further gauge demand, principally by way of soliciting possible applications. For example, respondents identified many applications for Feat, including: quantifying the food environment for local planning and making comparisons with other areas; local needs assessments; targeting education or skills-based individual-level interventions according to food environment characteristics; initiating/influencing cross-departmental obesity prevention strategies; and overall, using Feat alongside or in lieu of existing local food environment indicators in service of the public’s health. Four examples of map outputs from Feat that contain spatial data to support these applications, which include those showing information on electoral ward and address-level healthy and unhealthy food retail access (which can be quantified using the legend and histogram shown), and the extent to which this access has changed over time, are shown for central London in Figure 3.

Securing additional short-term funding allowed us to develop the Feat beta and cover data licensing costs, which enabled the launch of Feat freely online in July 2017. Feat’s launch was publicised by the UK Health Forum, and pre-launch, Feat was signposted for local authorities in PHE and the LGA’s *Strategies for encouraging healthier ‘out of home’ food provision*. The launch also received significant media coverage, including seven articles (comprising features and a spin-off interactive data visualisation) that emerged from active collaboration with the *Guardian* newspaper [35, 36]. As further evidence of initial impact, using web analytics we tracked 7000 page views, from 3250 visitors across 61 countries, within the first 5 weeks of Feat being publicly available. We continue to integrate user feedback to guide further iterations of the tool and new funding from the National Institute for Health Research is presently allowing us to build on our relationships with local government agencies and document case studies of Feat’s use in pursuit of public health impact.

## Comparing the tools: distinguishing features

The PCT and Feat are similar in that both display data on environmental risk factors related to obesity. However, they differ in some important ways. First, the tools differ in the fundamentals of what type of data they provide for visualisation. A key output of PCT is an estimation of the latent demand for cycling that *could be* tapped under various counterfactual scenarios. Alternatively, Feat illustrates quantitative profiles of the food environment, presently and historically. Thus, whereas Feat provides information about ‘*what is*’ and ‘*what was*’, PCT provides an illustration of ‘*what if*’.

Second, the tools differ in their origins with respect to policy. PCT was driven by an explicit policy imperative: to provide a framework and systematic evidence for guiding investment in cycling-related infrastructure across England. This ‘pull’ from policymakers provided important advantages, particularly in the form of funding, assurance of PCT’s place in the policy process, and a guaranteed customer through national level endorsement. In contrast, Feat was developed by researchers as a translational output of an empirical research programme, without any guarantees that the tool would be endorsed or even used by policy-makers and practitioners, and with only short-term funding. This more speculative ‘push’ model of Feat makes it distinct from the ‘pull’ model of PCT.

## Generalised learning experiences

From the development of PCT and Feat, we have identified the following common issues and challenges associated with the development of data visualisation tools by academics within higher education institutions. This is not intended to be an exhaustive list.

### Need for complex and varied skillsets and interdisciplinary working

Developing data visualisation tools for policy applications related to obesity is a complex, often lengthy process that goes well beyond the usual skillset possessed by academic researchers and departmental support in a higher education setting. Conceptualisation, development, implementation and dissemination of data visualisation tools requires expertise in multiple domains (see Feat case study). For the PCT, lacking expertise in web development meant that an external developer needed to be costed into the project. This may not always be possible, constituting a potential obstacle to progress. Even where skills exist, effective interdisciplinary working will require researchers to engage with and integrate the needs of others into their own ways of working. For example, web development of Feat imposed constraints on the architecture of the input geographic data, requiring the data to be transformed into GeoJSON format [37], previously unfamiliar to the researchers, and requiring new software. These new interdisciplinary ways of working inevitably require both time and patience, but are a critical investment in the long-term viability of most, if not all, interdisciplinary data visualisation tools.

### Costs for obtaining data and serving tool

Beyond the personnel and other costs associated with developing data visualisation tools, there are ongoing costs to maintaining and updating these tools. While many datasets are free to access and use, some, including data that are commercially-sensitive such as Ordnance Survey’s Points of Interest data (used in Feat), are not. These costs can be substantial and possibly prohibitive, and challenge traditional academic research funding channels, which are unlikely to be suitable for the purposes of creating tools. It may be difficult to monetise the use of tools, especially where the intended audiences are the public sector. Innovative funding models (e.g. freemium, spin-outs) may be a possibility, although testing can be risky, especially where the tool has previously been available for free.

### Identifying the customer and/or policy application

Research and data of interest to researchers are not necessarily of use to policymakers and practitioners for developing policy or guiding planning decisions. Likewise, data visualisation tools need to include capability that allows these users to answer their own questions of interest—not those of the researchers. This might include, for example, allowing users to manipulate real-world parameters, not just apply fixed hypothetical models. As the Chief Scientific Adviser for the Department of Health and Social Care has observed: ‘Models should, wherever possible, allow policymakers to vary assumptions’ [38]. A good understanding of the ‘market’ at which the tool is aimed is therefore necessary—and can be challenging to accurately establish without close existing links to policy customers. It does not follow that there must be a specific demand for the product itself, as the novelty of any tool is likely to be part of its appeal. But the tool should be aimed at a clear user-need that is not being met by current tools and data. Market demand can then be further pinpointed by early release and testing of a minimum viable product. This phase can also help align expectations between users and developers, and attract new potential users not previously identified.

### Establishing a strategy for effective dissemination

As with market research, dissemination is likely to be more effective if the marketing of these tools is part of a wider strategy of stakeholder engagement, which will have already opened up lines of communication between researchers and key users. The PCT and Feat both benefited, to different extents, from the existing dissemination methods and customer networks of their host organisations. A range of stakeholder engagement approaches are needed across social media, news media and interpersonal opportunities (face-to-face meetings, conference and policy forum presentations etc.). Dissemination is also an opportunity to spend social capital that has been built in earlier stakeholder engagement: requests for third-party endorsements, onward promotion by partner organisations and so on.

### Tracking engagement and demonstrating impact

Demonstrating early impact is likely to be a central part of making a compelling case for long-term funding. While engagement with tools can be tracked using straightforward web and social media analytics, these high-level statistics reveal relatively little about tool use and even less about onward application of lessons learned. More sophisticated methods of user tracking and analysis, such as A/B testing and multiple site versions, are more commonly used by commercial companies than academic researchers—often for simple reasons of cost and scale. And even these will be limited to capturing *any* time users spend directly with the tool Engagement with a tool (a *pathway* to impact) should not been seen as the same as any eventual *impacts* that arise following its use, which will be difficult to quantify and may take time to realise. Nevertheless, as part of the wider strategy of stakeholder engagement, it should be possible and sufficient to capture case studies of early use, which are important precursors to long-term public health impact. In this sense, the challenges of measuring the impact of these tools shares much with the general challenges of measuring the societal, economic and cultural impact of research: matching approaches to circumstances; considering feasibility and affordability; identifying suitable metrics and measures for both direct and indirect impacts; and applying emerging methods and lessons from what is a developing field [39].

### Long-term upkeep and user support

Many tools are only useful as long as they are kept to date. Where data costs are ongoing, this can prove challenging. Further costs include ongoing work to correct errors and other problems, and continued development to enhance functionality, for example by providing more data options. The online hosting of tools, data management and regular updating of data also carry costs. Some tools, for example the PCT, may also require the upskilling of practitioners in use and application of the tool in their local context, including how the tool could complement and/or build on existing local data and other resources. Options include online or face-to-face training, as well as ongoing one-to-one user support for specific applications.

## Conclusions

While the development of some data visualisation products can be externally ‘pulled’ through policy priorities, others arise because of entrepreneurial researchers working under permissive conditions. Conditions that best enable the development of more-speculative data visualisation tools include a departmental structure that encourages and facilitates communication and collaboration among interdisciplinary teams, together with time and resources to support production and testing. Either way, development of tools presents inevitable challenges, but as a novel pathway to impact, the speculative route may become more common.

As part of the UK’s ‘Impact Agenda’, universities and government research funding bodies are emphasising the need for academics to identify and develop pathways by which their research can have a societal impact [40]. Spatial data visualisation, such as those described here, are one way to address this need. Beyond their use within research programmes, data visualisations can be an important vehicle for reaching, and engaging with wider non-academic audiences [41]. We contend that these tools provide a novel gateway between researchers and a range of stakeholders, policymakers in local and national government in particular, who are increasingly seeking ways of accessing and using evidence to inform obesity programs and policies.
